# Flexibility to Change the Solution: An Indicator of Problem Solving That Predicted 9th Grade Students’ Academic Achievement during Distance Learning, in Parallel to Reasoning Abilities and Parental Education

**DOI:** 10.3390/jintelligence10010007

**Published:** 2022-01-27

**Authors:** Liena Hacatrjana

**Affiliations:** Department of Psychology, Faculty of Education, Psychology and Art, University of Latvia, Riga, Imantas 7. linija-1, LV-1083 Riga, Latvia; liena.hacatrjana@lu.lv

**Keywords:** academic achievement, COVID-19, distance learning, cognitive abilities, self-assessed skills, problem-solving, self-management skills, parental education

## Abstract

The relation between academic achievement and various measurements of cognitive abilities, problem-solving skills and self-managed learning has been established in the research before the COVID-19 pandemic and distance learning. The aim of the current research was to analyze the extent to which these aspects predicted the educational achievement of 9th grade students (mean age 15.4 years) during distance learning, when students had to do relatively more tasks independently, organize their daily learning and deal with problems on their own. Relations between self-assessed problem-solving skills, self-management skills, tests of reasoning abilities and the results of diagnostic tests in Mathematics and Latvian were analyzed for *n* = 256 and *n* = 244 students, respectively. The results show that: (1) diagnostic test results in Mathematics are best predicted by the parental education level, fluid nonverbal reasoning and verbal reasoning; (2) the best predictors for the results in the diagnostic test in Latvian are parental education, flexibility to change the solution, fluid nonverbal reasoning and verbal reasoning; (3) self-management cannot significantly predict the results of either of the two tests, although it correlates to the results of the tests in both Mathematics and Latvian; (4) only one of the aspects of problem-solving, flexibility to change the solution, can significantly predict results in diagnostic tests. The results confirm the significance of cognitive abilities as an important predictor of academic achievement, as well as the role of parents’ education level. The results also suggest that the flexibility to change the solution, an aspect of problem-solving, might play a role in students’ success in academic tests.

## 1. Introduction

During the COVID-19 pandemic, more than 1.6 billion children worldwide faced disruptions in face-to-face education, and many schools continued the educational process with distance learning (OECD 2020). To many students it was a new situation and their first experience with distance learning, bringing new challenges that could be considered as problems that needed to be solved daily. Different approaches to distance learning were applied in various countries around the world (Reimers and Schleicher 2020). In Latvia, a country among those with a high number of days of remote learning, mixed forms of learning were implemented (both synchronous and asynchronous) with online video lessons and with assigned tasks to be done individually at home (Ministry of Education and Science of Latvia 2020) indicating that the ability to work independently was demanded from students. Teachers were forced to swiftly adapt to using new technologies and using pedagogical techniques that worked online, but not all teachers were immediately ready for this: 25% of teachers reported that they had not organized any online lesson in the spring of 2020, when the first remote learning period was implemented, indicating that they had sent materials to students that had to be learned independently. Students were forced to learn on their own via online lessons with teachers or without direct online communication with teachers. During the first wave of pandemic, in spring 2020, about half of the students in Latvia reported that they lacked teachers’ explanations and motivation while at home and not in their classroom, and they felt stressed and unsure whether they would finish all tasks in time (Ministry of Education and Science of Latvia 2020), which indicates that an extra effort was asked of them.

The pandemic period and the distance learning have raised questions globally about which skills, abilities and other factors (e.g., external factors such as support from parents or teachers) are crucial for students to maintain their academic performance and well-being as much as possible during this time (Rosen et al. 2021; Hacatrjana 2021a), and some recent data have indicated a decrease in students’ academic performance that is probably due to the pandemic (Engzell et al. 2021). In this study, the focus is particularly on the individual aspects related to the students’ own skills and abilities to deal with the new situation. Students (most studies were performed in primary schools or in high schools) have reported in questionnaires that their ability to plan their time has helped them during distance learning, while also indicating feeling stress regarding the management of assignments on time and having insufficient planning skills, self-organization and management skills, which caused difficulties with remote learning (Scott et al. 2021; Ministry of Education and Science of Latvia 2020; Rogers et al. 2021; Hacatrjana 2021a). This means that during the pandemic and distance learning many students were aware that they lacked some skills to learn effectively. In addition, the pandemic situation and the unprecedented distance learning were essentially a new challenge for most students, and thus they needed to adapt to the new situation and use their problem-solving skills to cope with it and to study independently in a successful manner. In Latvia, a change in the curriculum was recently introduced in schools, and problem-solving skills and self-regulated learning skills are among the transversal skills that are deemed important for students in Latvia and that should be further developed at schools (Cabinet of Ministers Republic of Latvia 2018). In addition, previous PISA results have indicated that the results on problem-solving skills in this country are below the OECD average (OECD 2017). During the pandemic a study with high-school students in Latvia showed that students with higher self-reported problem-solving skills were less stressed about distance learning (Hacatrjana 2021a). All these previously obtained results suggest that problem-solving skills and self-management skills are essential for students to adapt to new circumstances and to maintain their academic achievement during distance learning in the COVID-19 pandemic.

The close link between various measurements of academic achievement and indicators of cognitive abilities has been well established in the research literature (way before the pandemic), proving that cognitive abilities predict academic achievement to a great extent (e.g., Frey 2019; Kampa et al. 2021). Indicators of other skills show a relation to academic achievement as well, both with GPA and SAT tests. For example, problem-solving tests predict academic achievement (Greiff et al. 2014; Greiff et al. 2013). Self-management indicators, mostly assessed with self-assessment type questionnaires, are also related to academic achievement (Pintrich et al. 1993; Zimmerman and Martinez-Pons 1988; Abd-El-Fattah 2010; Veenman et al. 2014). Problem-solving skills (assessed via various methodological approaches) and cognitive ability also show interrelationships (e.g., Chuderski and Jastrzębski 2018; Kretzschmar et al. 2017; Ellis et al. 2021). In general, these results indicate that there is a set of cognitive abilities and additional skills which together can predict a student’s learning performance in regular learning settings. Given the differences from the usual learning environment and the format in which most research has taken place in this field, it is important to explore the extent to which cognitive abilities and additional skills—problem solving and self-management skills—predict student performance during distance learning in the pandemic. The aim of the current research is to examine whether problem-solving skills and self-management skills, in parallel with tested cognitive abilities, can predict the results of 9th grade students’ diagnostic tests (an indicator of academic achievement) during the distance learning period due to the COVID-19 pandemic, as it is discussed that both these skills are important when studying independently.

### 1.1. Problem-Solving Skills and Self-Management Skills: Important for Studying Independently

Assuming that problem-solving skills and self-management skills are important for students during distance learning (Hacatrjana 2021a), it is useful to unravel them in more detail in the context of this study. Regarding the research of problem-solving skills, there are several approaches in Psychology that differ based on their theoretical framework and methodology (e.g., Frensch and Funke 1995; Heppner and Petersen 1982; OECD 2013, and others). In addition, they are also defined as important skills in the education field in many countries, which seek to teach them to students (e.g., in Latvia, Cabinet of Ministers Republic of Latvia 2018). Most researchers in Psychology state that problem-solving consists of several underlying processes, often similar to the original ideas of George Polya proposed many years ago: (1) understanding the problem, (2) devising a plan, (3) carrying out the plan and (4) looking back (Polya 1957). One of the modern approaches that focuses on studying the abilities of complex problem solving empirically defines that there are two main processes underlying problem-solving: (1) knowledge acquisition and (2) knowledge application (Fischer et al. 2012). In the global PISA educational assessment problem-solving is considered to consist of several processes: (1) exploring and understanding; (2) representing and formulating; (3) planning and executing; (4) monitoring and reflecting; and reasoning is used during the process of problem-solving (OECD 2013). Other approaches focus on the self-assessment of the attitudes and experience in problem-solving (for example, Heppner and Petersen 1982), and problem-solving processes in specific fields—for example, in Mathematics (Verschaffel and De Corte 1993). What the different approaches have in common is that the aspects of problem-solving skills are applied when facing a situation or a task which cannot be solved by an automated action, and often a clear and good solution is not immediately known, especially when facing new problems and situations.

Problem-solving skills in the context of the current research are defined by the author as a set of skills, habits and operations that help individuals (e.g., students), when facing a new task or problem, to successfully explore and understand the key concepts involved in the problem, to be able to come up with possible solutions, to implement a solution, to be able to realize if the solution is not appropriate and react accordingly (thus being flexible in the process of solving the problem), and to evaluate the result and process of problem solving. Problem-solving skills are here operationalized by the self-assessment of two aspects of problem solving: (1) Solution development and evaluation and (2) Flexibility to change the solution (see Methods section), indicating that during problem solving it is important to come up with possible solutions and evaluate the result afterwards, as well as being flexible to change the chosen solution strategy if it is not suitable. Flexibility is a variable also studied in the field of mathematical problem solving and it is related to academic achievement (e.g., Hästö et al. 2019). It includes the knowledge of various possible strategies and the ability to implement the appropriate option. These ideas from the field of mathematics could be transferred to problem solving in general, meaning that flexibility in problem solving indicates the ability to choose between the options a person can think of and to apply the most appropriate solution during the process of problem solving. 

Self-management is a process that is involved in self-regulated learning (Zimmerman 2008), an important concept in modern educational approaches, and especially crucial during distance learning. Self-regulated learning is a very broad concept that consists of several important aspects. Both metacognitive processes and the ability to organize oneself practically are important for a person to become good at self-regulated learning. During the learning process students use their metacognition to proactively think, perform and self-reflect (Carter et al. 2020), which is assumed to lead to good self-management. According to the model by Garrisson (1997), self-management and self-organization are an important part of the broader concept of self-regulated learning, and they relate to how the activities associated with learning are carried out and controlled, such as how all the necessary resources are managed.

In the current research, self-management skills are defined by the author as a set of skills and habits necessary to (1) successfully organize one’s tasks, time and resources and (2) be able to understand conceptually and clearly what has to be done in a certain period of time and why it has to be done (motivational aspect). Thus, self-management is here focused mainly on one’s practical organization, during the process of learning, also keeping the motivational aspect and focusing on the goal, as in the original ideas by Garrisson (1997).

The rationale of the current study is that it is important to assess how problem-solving skills and self-management skills are related to academic outcomes during unprecedented events such as the COVID-19 pandemic to make conclusions and develop further hypotheses about these skills in education (as a part of the curriculum). If these skills are shown to be important predictors during the pandemic, then we can further assume that they are indeed important skills to develop at schools because they might help students to adapt to any other unprecedented events that may come in the future.

### 1.2. Methodological Considerations Regarding the Relation between Self-Assessed Skills and Test Results

The analysis of the relationship between self-management, problem-solving skills, cognitive abilities and academic achievement should consider the research methodology—whether the skills are assessed by tests or by self-assessment methods. Researchers have proven that computer-based problem-solving skill tests have shown high correlations with cognitive abilities for high-school students (e.g., Kretzschmar et al. 2017) and a high ability to predict academic performance in primary and high school (e.g., Greiff et al. 2013). Some studies show that self-assessment measurements for problem solving also tend to show statistically significant relationships with cognitive test scores in the high-school population (e.g., Nota et al. 2009), and that self-assessed self-directed learning skills are related to academic performance, measured in an undergraduate sample (Tekkol and Demirel 2018).

When assessing the suitability of the self-assessment methods in educational settings in general, it becomes clear that research results on self-assessment accuracy are not consistent (Brown and Harris 2013), with some previous research showing that students’ self-assessments correlate with the grading by teachers in schools (Sanchez et al. 2017). However, in some research, gender and other differences are reported regarding the accuracy of self-assessment—for example, the tendency for undergraduate students with higher marks to rate themselves more precisely compared to students with lower marks (González-Betancor et al. 2019; Kim et al. 2010). In a study with 9th grade students from Latvia, it was found that self-efficacy in mathematics was higher for boys (Kvedere 2014). Self-estimates of intelligence and test scores are low to moderately correlated in research with various samples (Rammstedt and Rammsayer 2002; Furnham and Grover 2020). Research also shows that undergraduate students with lower performance are more likely to overestimate themselves but are aware of the possible inaccuracy, and students with higher performance are more accurate in their self-evaluation (Miller and Geraci 2011). In general, the importance of self-assessment as a means for development of skills is discussed in the literature, and there is a tendency to increasingly include self-evaluation in the process of learning (Andrade 2019; Vasileiadou and Karadimitriou 2021). Research results indicate that there might be flaws in the precision of the self-assessment of one’s skills, especially if the self-assessment affects the final mark (Andrade 2019), which was not the case in the current study. The author of this research used several methods to minimize the possible flaws of self-assessment: first, the participation in the study was anonymous, and students were encouraged to answer truly to themselves, not thinking about any “right or wrong answers”; and second, the questionnaire used in the study included indicators of specific operations that characterize problem solving and self-management that were clearly defined, and students had to evaluate how often they performed such activities. Thus, the ratings were based on the frequency of an action, not on the agreement with a statement. 

### 1.3. Academic Achievement Assessments during Distance Learning in the COVID-19 Pandemic

Another important issue that must be elaborated regarding the topic of this article is the practice and challenges of measuring academic achievement during the pandemic and the distance learning that was implemented in most countries as a response to the spread of the SARS-CoV-2 virus (OECD 2020). During this complicated time, there were different approaches implemented regarding students’ assessment (Kuhfeld et al. 2020; Thorn and Vincent-Lancrin 2021). For example, summed scores by teachers were implemented in Ireland (Doyle et al. 2021). In Latvia the traditional exams at the end of primary school after the 9th grade have always been important and might determine one’s chance of getting into a high school. However, during the COVID-19 pandemic a decision was made by the government that the usual exams after the 9th grade would be replaced with “diagnostic assessment tests” in the same taught subjects (mandatory for Mathematics and Latvian, optional for English, Sciences and History) (Cabinet of Ministers Republic of Latvia 2021). The diagnostic tests would not affect students’ final grading at the end of primary school and further opportunities to join a high school or another schooling option. These tests would be similar in their content and level of difficulty to exams, and all 9th graders in Latvia took the same tests at the same time period, thus making this score appropriate for directly comparing the results of students in different schools. The results of these diagnostic tests were used in the current study as an indicator of the academic achievement of students.

### 1.4. Focus of the Current Article

This paper aims to determine the best predictors of diagnostic test results and the extent to which problem-solving skills and self-management skills were able to predict the results of diagnostic tests during distance learning in the pandemic situation, given that they were (in this case) determined with self-assessment methods, along with cognitive abilities that were assessed with test tasks and parental education level. As discussed before, academic achievement is proved to be closely related to indicators of cognitive abilities (e.g., Frey 2019; Kampa et al. 2021), and therefore it is assumed that in the current study the indicators of cognitive abilities should also significantly predict academic achievement. The significant relation between parental education level and academic achievement is also established in previous research (e.g., Idris et al. 2020). Thus, it is assumed that parental education should be an important predictor for academic achievement also in the current study. A recent study confirms the importance of parental education to grades, but it is concluded that intelligence is a more important predictor than the whole socioeconomic status measurement (Flores-Mendoza et al. 2021). It is assumed that problem-solving and self-management skills could play an important role in how well students were able to maintain their academic performance even during distance learning (Hacatrjana 2021a). This means that, hypothetically, if a student has good grades and high problem-solving skills, then he or she should also be able to deal well with studying in a new, unprecedented problem situation—distance learning. It is similar with the self-management and self-organization skills—if the student is doing well at school and has these skills highly developed, then it is easier for him or her to cope with distance learning, and vice versa—if a student has generally good grades, but he or she lacks self-management skills or problem-solving skills, then the distance learning process could have a greater impact on a student’s performance, and academic achievement may be lower due to the lack of the skills to organize oneself and deal with problems.

The main question is: if we assume that problem-solving skills and self-management skills are indeed important to successfully cope with distance learning, will it show in the results of students’ academic outcomes during this period? The aim of the current study is to examine this assumption, taking into account that these skills were assessed with self-assessment methods. The research questions posed in the current study are: (1) What are the best predictors of the results of students’ diagnostic tests at the end of 9th grade during the distance learning in the COVID-19 pandemic? (2) To what extent do the self-assessed problem-solving skills and self-management skills predict the results of diagnostic school tests of the mentioned population?

## 2. Materials and Methods

### 2.1. Sample

The data of *n* = 652 students in the 9th grade from general education schools in Latvia (359 females, 293 males), aged 14 to 17 years (M = 15.41, SD = 0.53), were gathered; the sample size used in the regression analysis is smaller due to a smaller amount of some of the indicators obtained from schools; in such cases, the precise amount of analyzed cases is reported within the results. 

### 2.2. Measurements

(1) Problem-solving skills were evaluated with a problem-solving questionnaire, a self-assessment method with 10 items comprising two scales, that were named: (1) Solution development and evaluation (6 items) and (2) Flexibility to change the solution (4 items), that originally showed an internal consistency of, respectively, α = 0.79 and α = 0.71 (Hacatrjana 2021b). Each item had to be rated on a scale from “Never” to “Always” (0 to 5 points) based on how often a student performed the mentioned activity (item examples: “When solving a situation or doing a task, I change my solution if I understand that it is not appropriate”, “When I have finished a task, I think about what worked well and what didn’t.”). The scale “Flexibility to change the solution” is significantly correlated to the results of nonverbal and verbal reasoning tests (r = 0.22 and r = 0.25, *p* < 0.01, respectively), indicating its validity, but statistically significant correlations are not found with the scale “Solution development and evaluation”. Both scales of the questionnaire are significantly correlated (r = 0.46, *p* < 0.01).
(2) Self-management skills were assessed with the self-management questionnaire that is used for the purpose of self-assessing students’ skills to manage and organize themselves and their learning. It consists of six items (for example, “I write down all the tasks in a certain place”, “If I lose motivation at some point, I remind myself why it was important for me to do it”), that originally showed an internal consistency of α = 0.77. Each item had to be rated on a scale from “Never” to “Always” (0 to 5 points) based on how often a student performed such an action (Hacatrjana 2021b). In the current study the Self-management scale is negatively correlated to the students’ self-evaluations of their perceived difficulty to deal with distance learning (r = −0.12, *p* < 0.01), indicating the validity of the scale.
(3) Fluid nonverbal reasoning was measured with a short version (10 items) of the Sandia Matrices test (see Harris et al. 2020; Matzen et al. 2010), that assesses reasoning abilities with typical figural matrices tasks where one has to understand the patterns in a set of drawings and choose the most appropriate answer (a drawing that continues the pattern) from eight answer options. The internal consistency of the test, measured with Chronbach’s alpha, was α = 0.72. Each answer is rated with 0 or 1 point.
(4) Verbal reasoning was assessed with a short version of the Verbal analogies test (10 items) that has been previously developed and used in the research with students (Kretzschmar et al. 2017). In the test, one pair of words and the first word of the second pair is given (for example, “snow—to ski” and “ice—…”), and the participant has to understand the type of relationship for these words and write an answer to the second pair of words. The internal consistency of the test, measured with Chronbach’s alpha, was α = 0.81. Each answer is rated with 0 or 1 point.
(5) Academic achievement was measured by gathering several indicators from schools: results in diagnostic tests at the end of the 9th grade in Mathematics, Latvian and English. The tests were taken by students online during the pandemic, and each test was administered on a specific date set by the state. The test was exactly the same for all students in the country. It must be noted that not all students took all of the tests (some are optional, e.g., English), and not all schools provided the researcher with the necessary anonymized data; thus, the amount of available data is smaller for these test results compared to the data from other measurements. The exact amount of data analyzed is shown further in the results section. In each test a student can get from zero to a maximum of 100 points.
(6) Additional questions on experience and attitudes during distance learning were asked to students: for example, to rate their perceived difficulty to deal with the distance learning situation, to assess whether the technological means available to them were sufficient for studying. Students had to rate these questions on a Likert scale with 0 to 5 points. It was also asked if a student had been to an individual consultation with a teacher (individual face-to-face consultations were allowed as an exception at that period of time for students facing difficulties).
(7) Demographic questions were asked: gender, age, the level of parental education (from “1-Finished Primary school” to “6-Doctoral degree”). Each student wrote the individual code that was assigned by the school for each student to ensure confidentiality.

### 2.3. Procedure

The data collection was carried out in close collaboration with each participating school, in two rounds: (1) Students filled out the tests and questionnaires online. Students from each class joined a specifically scheduled online lesson on a platform typically used by the particular school in the period of distance learning (platforms “Microsoft Teams” and “Zoom” were most commonly used). Students were first informed about the study and instructed, and then they went to the testing site, on the internet, where they completed surveys and tests in 40–50 min. A link to the tests was given to the students at the beginning of the testing. The instructor remained connected to the online lesson to answer technical questions, if any came up. Pupils were asked to talk and ask only questions about technical uncertainties during the test, but not to communicate for other reasons so as not to disturb others. (2) The school representative compiled academic performance indicators: the results of diagnostic tests that were administered as a final assessment at the end of the 9th grade (primary school). Data were collected in an anonymized form, each student having their own code. The codes were assigned by the school based on the system recommended by the researcher (using letters + numbers denoting school, class and student number). The student was informed of his or her code shortly before the online testing, and then the student wrote this code on the testing site when starting the tests. The same code for each student was used when the academic achievement indicators were administered and sent to the researcher. Before the research started, each school had been informed about the aims and procedure of the research, and an informative letter to the parents was sent out by the school to allow for the participation in the study.

### 2.4. Data Analysis

To answer research questions, the following statistical analysis methods were used: multiple regression analysis, *t*-test and Spearman’s correlation coefficients. The data were analyzed with statistics package SPSS version 22.

## 3. Results

First, the descriptive statistics of the indicators measured are presented (see Table 1). As we can see in Table 1, the amount of data regarding the results of the diagnostic test in English is not sufficient to perform further analyses.

The internal consistency measured by Cronbach’s alpha was calculated for Self-management (α = 0.76), Problem-solving: scale Solution development and evaluation (α = 0.77) and Problem-solving: scale Flexibility to change the solution (α = 0.70), showing appropriate levels in the current sample. The data show that students come from classrooms with 9 to 34 students per class (M = 22.21; SD = 4.71), and such a variety is typical in Latvia, if students from smaller schools are compared to students from large schools. 

Table 2 shows the Spearman’s correlation coefficients between the measured indicators. The results of the diagnostic test in Latvian have a significant relation to Parental education level, Fluid nonverbal reasoning and Verbal reasoning, the Self-management scale and both scales of Problem solving: Solution development and evaluation and Flexibility to change the solution. The results of the diagnostic test in Mathematics show statistically significant correlations with Parental education level, Fluid nonverbal reasoning and Verbal reasoning, the Self-management scale and one scale of Problem-solving: Flexibility to change the solution, and are negatively correlated to the subjectively felt difficulties in dealing with distance learning. Both diagnostic tests (Mathematics and Latvian) show a significant interrelation, with r = 0.62. No significant correlation was found between the age of participants and the result of the diagnostic test in Mathematics (r = −0.05, *p* = 0.39) or the results of the diagnostic test in Latvian (r = −0.03, *p* = 0.64), and thus age would not be further included in the regression analysis. No significant difference was found between the gender of participants and the result of the diagnostic test in Mathematics the with statistical *t*-test analysis (t = 0.38, *p* = 0.71), with M = 61.81, SD = 22.65 for girls and M = 60.89, SD = 21.84 for boys. However statistically significant gender differences were found in the results of the diagnostic test in Latvian (t = 4.12, *p* = 0.00), with higher results for girls (M = 64.29, SD = 15.74) and lower results for boys (M = 57.15, SD = 15.43), indicating that gender should be included in the regression analysis.

A multiple regression analysis was performed separately for the results of the diagnostic tests in Mathematics and Latvian to examine which were the best predictors. First, the regression analysis for the diagnostic test in Mathematics (DM) was performed. The indicators that correlate to the results in DM were included as independent variables in the hierarchical regression analysis (see Table 3). In the first step, the level of parents’ education was entered, and explains 13% of the variation in the results of DM. Further, the problem-solving aspect Flexibility to change the solution was included and adds 4% to the variation. Self-management is not a statistically significant predictor of the results of DM. However, Fluid nonverbal reasoning and Verbal reasoning complement the additional 10% and 7%, respectively, to predict the variance in DM. The results indicate that higher students’ Parental education level, Nonverbal reasoning, Verbal reasoning and Flexibility to change the solution led to higher results in the diagnostic test in Mathematics. It can be seen, however, that in Step 5, where all other measurements are included, both of the self-assessed measures (Self-management and Flexibility to change the solution) do not show statistically significant results (β = 0.11 and β = −0.04, respectively). When a simple regression was calculated, entering only the indicator Flexibility to change the solution as an independent variable, it showed that this indicator alone could explain 7% of the variation in DM (R^2^ = 0.07, F = 24.17, *p* = 0.000; B = 1.60, SE = 0.33, β = 0.26, *p* = 0.000).

Secondly, the indicators related to the results of the diagnostic test in Latvian (DL) were included as independent variables in the multiple regression analysis (see Table 4) to find out which are the best predictors of DL.

It can be seen in Table 4 that, in the first step, gender explains 4% of the variance in DL (higher for girls) and, in addition, parental education level explains another 7% of this variance. Together, they explain 11% of the variance. Further, the problem-solving aspects Solution development and evaluation and Flexibility to change add an extra 2% and 8%, respectively. As in the regressions performed for the DM, Self-management does not predict the results of DL in a statistically significant manner. In Step 6 and Step 7 Fluid nonverbal reasoning explains an additional 7%, and Verbal reasoning explains an additional 10% in the variance of DL. In Step 7 the indicators Self-management and Solution development and evaluation, as well as gender, are not statistically significant. When the following variables were entered as independent variables in a separately performed multiple regression analysis—Parental education level, Flexibility to change the solution (Problem-solving), Fluid nonverbal reasoning and Verbal reasoning—it was shown that, together, they could predict the DL test results and explained 36% of the variance (R^2^ = 0.36, F = 34.05, *p* = 0.000). 

Returning to the research questions stated in this study, it can be concluded that (1) the best predictors for the results in DM are Parental education, Fluid nonverbal reasoning and Verbal reasoning; (2) the best predictors for the results in DL are Parental education, Flexibility to change the solution (an aspect of problem solving), Fluid nonverbal reasoning and Verbal reasoning; (3) Self-management cannot significantly predict the results of DM or DL, although it correlates to the results of both DM and DL; (4) only one of the aspects of problem solving, Flexibility to change the solution, is predictive of the results in diagnostic tests.

## 4. Discussion

One of the aims of the current research was to determine the best predictors of results in school diagnostic tests at the end of the 9th grade (considered as important indicators of academic achievement) during the distance learning due to the COVID-19 pandemic. The pandemic was an unprecedented problem, during which distance learning was introduced for students who had never learned in such a way. For many students, it was a new situation, posing many new problems (e.g., planning one’s time, motivating oneself and lack of regime) (Hacatrjana 2021a). Thus, it was assumed that problem solving and self-management skills would be necessary to effectively learn independently and reach academic goals during this time, in parallel to cognitive abilities and parental education level, that have both proved to be important predictors of academic achievement (e.g., Flores-Mendoza et al. 2021).

The results of the current study show that there are some differences regarding the predictors of the results of diagnostic tests in different fields of study—Mathematics and Latvian. The best predictors for the diagnostic tests in Mathematics of 9th graders are their cognitive abilities (in this case—fluid nonverbal reasoning and verbal reasoning) and parental education level, explaining altogether about a third of the variance in the Mathematics test. Only one aspect of problem solving—the flexibility to change the solution—showed an additional contribution that was statistically significant, when analyzed separately. As to the results of the diagnostic test in Latvian, the level of parents’ education, the flexibility to change the solution (one aspect of problem-solving), fluid nonverbal reasoning and verbal reasoning have a predictive value. Together, these variables can explain more than a third of the variance in the results of the diagnostic test in Latvian.

A conclusion which can be generalized to the tests in both subjects (Latvian and Mathematics) is that a more important role is played by cognitive abilities (in this case fluid nonverbal and verbal abilities) in comparison to self-assessed indicators of skills. It is argued that students might give socially desirable answers to self-report questions or might not be precise enough in evaluating their abilities. Moreover, previous studies have shown that cognitive abilities assessed with tests are indeed the strongest predictor of academic performance (e.g., Demetriou et al. 2019; Frey 2019; Kampa et al. 2021), though the contribution is lower for older students compared to younger students. Conway and Hao (2020) argue for the need for precise methodologies if we want to assess the relation between non-cognitive factors and SAT scores. The authors argue that cognitive test scores typically explain at least half of the variation in SAT tests, if cognitive measurements have been adequately selected and cover a full range of abilities. In the current study the cognitive abilities did not explain such a large proportion of the variance, possibly due to this very reason.

The results presented here also showed the importance of parental education level to the school test results. Having parents with a higher level of education predicts higher results in academic achievement for students, and, as other research shows, it might be even more important during the pandemic (Easterbrook 2021). The tight relation between parental education and academic achievement is already established in previous studies and discussed in the literature (e.g., Idris et al. 2020; O’Connell and Marks 2021). It might be explained not only by the level of abilities, but also by higher parents’ involvement and valuing education as important in life based on their own experience (Lara and Saracostti 2019). During distance learning, parents’ involvement might have played an even larger role, and research shows that parental knowledge and comprehension of education, as well as proficiency in technology, was related to several indicators, such as the encouragement of an effective use of technology for education (Dimopoulos et al. 2021). 

The second aim of the study was to examine the extent to which self-assessed problem-solving skills and self-management skills could predict the results in diagnostic tests of 9th graders during distance learning. Two aspects of problem solving were measured: the flexibility to change the solution and solution development and evaluation, and a total score of self-management was obtained. Compared to cognitive abilities and parental education level, these skills have a much smaller influence on the test results. Nevertheless, one aspect of problem solving in particular—the flexibility to change the solution—can explain a relatively small but statistically significant proportion of the variance of the test results. This aspect of problem-solving is briefly discussed below. 

The flexibility to change the solution is an important aspect of problem solving (Hacatrjana 2021b) and was significantly predictive of the results in the diagnostic test in Latvian. It was also predictive of the diagnostic test results in Mathematics when analyzed separately. But when other variables are included into the regression, the significance of this indicator drops, and other variables—nonverbal and verbal reasoning, as well as parental education—become the most important predictors. Why is the flexibility to change the solution important to get better results in tests and why, in the current research, does it turn out to be more important than the ability to come up with solutions and evaluate them? The flexibility to change the solution might be a crucial aspect to successfully solve problems or tasks, providing that an individual is able to, first, detect if something is wrong in the solution; secondly, make a decision to start over or change something in the solution; and third, come up with an alternative or a new way to do the task and execute it. It might be related to the ability to switch between ideas and possible solutions, and not to get stuck on the first solution that has come to mind. In the mathematics, the term “flexibility” characterizes the ability to choose between several solving options (meaning that the student is aware of various approaches and is able to implement them when necessary) and is also related to academic achievement (Hästö et al. 2019). The results of the current study might also be explained by the fact that the Flexibility to change is significantly correlated to both cognitive tasks: Fluid nonverbal reasoning and Verbal reasoning, while the other aspect of problem solving—Solution development and evaluation—shows weaker correlations with these tasks. The fact that the Solution development and evaluation aspect is less related to the diagnostic test results, compared to the Flexibility to change the solution, is worth studying further, to examine if the flexibility in one’s actions during a problem- or task-solving process is crucial to successful problem solving in general, as these results suggest, and to what extent flexibility is related to cognitive abilities and might be taught as a skill and an attitude.

Another important finding in the current study is that the self-management skills failed to show statistically significant results in regression analysis to predict the results in the Mathematics and Latvian tests, though self-management skills were correlated significantly to the results of these tests. This contradicts previous research showing a significant relation between self-management or other aspects of self-regulated learning and indicators and academic achievement (Pintrich et al. 1993; Zimmerman and Martinez-Pons 1988; Abd-El-Fattah 2010; Veenman et al. 2014), and the results from a study where students revealed the importance of self-management skills during distance learning (Hacatrjana 2021a). How could these results be explained, considering the importance of self-management (and self-regulated learning as a broader term) in education? One of the explanations is that these studies vary in the methods used and the conceptualization of terms, such as self-management in learning or self-regulated learning. Another explanation is that other indicators measured in this study are just stronger predictors, having tighter correlations to the test results, and thus statistically self-management skills are left below the line. This could also be due to the conceptualization of the self-management construct that was measured in the current study. It covers the actions of planning and organizing one’s learning process and physical settings and maintaining the motivation to do the school tasks but does not cover broader aspects of self-regulated learning, such as implementing learning strategies. One explanation for these results might be that the skills included in this concept are indeed important for focusing on the studies and an accurate approach to learning on a daily basis and managing daily learning tasks, but they are not sufficient to increase the level of performance in the academic tests. 

Overall, the currently presented results reveal that a relatively little contribution is made by the problem-solving and self-management skills, assessed with self-assessment methods, to the results of school tests during the pandemic. Yet, an important aspect of problem-solving skills—flexibility to change the solution—does make an additional contribution and explain the variance of the test results, especially in the Latvian test. There are no comparison data on students’ problem-solving or self-management skills before the pandemic. Nevertheless, the importance of developing students’ skills and habits to effectively deal with problems and obstacles should not be neglected, as it is previously proven that some aspects of problem solving can be successfully developed in the classroom (Verschaffel et al. 1999). While reasoning abilities are crucial for doing well in the diagnostic tests, it is also important to teach students the skills and strategies to apply when facing new or complicated tasks, so that they can think of solutions, implement them, and make the decision to change something in the solution if it turns out to be inappropriate.

### Limitations

For some measurements (scores in the diagnostic tests), the data were not fully provided by the schools, mostly due to lack of capacity of workforce resources (the data needed to be coded to anonymize students’ names). This led to a smaller amount of data used for such measurements as diagnostic tasks. The study would have benefited if a wider variety of cognitive measures had been used to examine if an even larger contribution would be made to explaining the variance in students’ test results. The level of students’ assessment of their skills before the pandemic is not known, and thus a conclusion on the dynamic of these skills before and during the pandemic cannot be drawn—only conclusions on the relation between skills and academic achievement during the pandemic. The study involved the self-assessed measurements that were previously discussed, and it would have benefited if the teachers’ ratings of students’ skills (for example, self-management) had been used to enhance the validity of these measurements. However, during distance learning, the teachers could not directly observe how a student is organizing his or her daily learning process, and the ratings would also be based on their previous experience.

## 5. Conclusions

Several indicators that might have predicted the results of diagnostic tests in Mathematics and Latvian at the end of primary school (9th grade) during the unprecedented COVID-19 pandemic and the corresponding distance learning were analyzed in this study: cognitive abilities, verbal reasoning and nonverbal reasoning, self-reported problem-solving skills and self-management skills and parental education level. The most important predictors for the test results were cognitive abilities and parental education level, and only one aspect of problem solving: the flexibility to change the solution. Self-assessed problem-solving skills and self-management skills did not play such an important role in predicting the results in the diagnostic tests taken during distance learning, as would be expected. We can speculate that self-management skills were probably important in the daily management of one’s learning during distance learning, as shown by previous research, but they were not decisive to reach higher academic results in tests at the end of the 9th grade. One aspect of problem-solving—the flexibility to change the solution—contributed to the results in diagnostic tests, especially in Latvian (the native language), indicating that this might be an important set of skills and attitudes for students to develop to successfully deal with school tasks. Based on this analysis, conclusions can be drawn about the importance of these skills to maintain academic achievement when students are facing new situations. It can be further assumed that it is justified to teach problem-solving skills as part of the curriculum, as they might help students adapt to other unprecedented events in the future.

## Figures and Tables

**Table 1:** Descriptive statistics of the indicators measured in this study.

Measured Indicator	N	Min	Max	M	SD
Parental education level	630	1	6	3.44	1.24
Age of the student	655	14	17	15.41	0.53
I have felt difficulties dealing with studies during distance learning	659	0	5	3.11	1.32
The technological means available to me at home are sufficient to study remotely	659	0	5	4.36	0.97
Fluid nonverbal reasoning	534	0.00	10.00	4.96	2.63
Verbal reasoning	615	0.00	10.00	5.81	2.80
Self-management scale	647	1.00	30.00	16.31	6.11
Problem-solving: scale *Solution development and evaluation*	649	0.00	30.00	14.91	5.20
Problem-solving: scale *Flexibility to change the solution*	649	0.00	20.00	12.85	3.45
Diagnostic test in English	77	53.00	100.00	85.64	10.52
Diagnostic test in Latvian	330	14.29	99.09	61.17	15.99
Diagnostic test in Mathematics	347	10.67	100.00	61.44	22.25

**Table 2:** Correlations between the measured indicators for the students in the 9th grade.

		1	2	3	4	5	6	7	8	9	10
1. Parental education level	Correlation Coefficient	1.00									
	n	630									
2. I have felt difficulties dealing with studies during distance learning	Correlation Coefficient	−0.11 **	1.00								
	n	630	659								
3. The technological means available to me at home are sufficient to study remotely	Correlation Coefficient	0.06	−0.05	1.00							
	n	630	659	659							
4. Diagnostic test in Latvian	Correlation Coefficient	0.25 **	−0.09	−0.01	1.00						
	n	317	330	330	330						
5. Diagnostic test in Mathematics	Correlation Coefficient	0.32 **	−0.13 *	0.04	0.62 **	1.00					
	n	332	347	347	330	347					
6. Fluid nonverbal reasoning	Correlation Coefficient	0.16 **	−0.05	0.02	0.35 **	0.38 **	1.00				
	n	510	534	534	270	282	534				
7. Verbal reasoning	Correlation Coefficient	0.17 **	−0.04	0.04	0.52 **	0.49 **	0.45 **	1.00			
	n	588	615	615	312	326	501	615			
8. Self-management	Correlation Coefficient	0.08	−0.12 **	0.11 **	0.21 **	0.13 *	−0.023	0.012	1.00		
	n	619	647	647	329	345	526	609	647		
9. Solution development and evaluation	Correlation Coefficient	0.09 *	−0.02	0.11 **	0.13*	0.06	−0.07	−0.01	0.45 **	1.00	
	n	621	649	649	329	345	528	611	647	649	
10. Flexibility to change the solution	Correlation Coefficient	0.18 **	−0.01	0.13 **	0.34 **	0.25 **	0.22 **	0.25 **	0.37 **	0.46 **	1.00
	n	621	649	649	329	345	528	611	647	649	649

* *p* < 0.05; ** *p* < 0.01.

**Table 3:** Regression analysis of the result of the diagnostic test in Mathematics (*n* = 256) with independent variables: Parental education level, one scale of Problem-solving: Flexibility to change the solution, Self-management, Fluid nonverbal reasoning and Verbal reasoning.

	B	SE	β	F	R^2^	ΔR^2^
**Diagnostic test result in Mathematics**						
Step 1				38.52 **	0.13	0.13
Parental education	6.27	1.01	0.36 **			
Step 2				11.96 **	0.17	0.04
Parental education	5.67	1.00	0.33 **			
Flexibility to change the solution	1.21	0.35	0.20 **			
Step 3				0.05	0.17	0.00
Parental education	5.69	1.01	0.33 **			
Flexibility to change the solution	1.24	0.39	0.21 **			
Self-management	−0.05	0.22	−0.02			
Step 4				34.59 **	0.27	0.10
Parental education	5.50	0.95	0.32 **			
Flexibility to change the solution	0.80	0.37	0.13 *			
Self-management	−0.09	0.20	−0.03			
Fluid nonverbal reasoning	2.75	0.47	0.33 **			
Step 5				24.68 **	0.34	0.07
Parental education	4.87	0.92	0.28 **			
Flexibility to change the solution	0.66	0.35	0.11			
Self-management	−0.14	0.19	−0.04			
Fluid nonverbal reasoning	1.69	0.50	0.20 **			
Verbal reasoning	2.31	0.47	0.29 **			

* *p* < 0.05; ** *p* < 0.01.

**Table 4:** Regression analysis of the result of the diagnostic test in Latvian (*n* = 244) with independent variables: Gender, Parental education level, Solution development and evaluation and Flexibility to change the solution, Self-management, Fluid nonverbal reasoning, Verbal reasoning.

	B	SE	β	F	R^2^	ΔR^2^
**Diagnostic test result in Latvian**						
Step 1				9.50 **	0.04	0.04
Gender	−6.31	2.05	−0.19 **			
Step 2				20.39 **	0.11	0.08
Gender	−6.47	1.96	−0.20 **			
Parental education	3.45	0.76	0.27 **			
Step 3				4.18 *	0.13	0.02
Gender	−6.04	1.97	−0.19 **			
Parental education	3.27	0.76	0.26 **			
Solution development and evaluation (Problem solving)	0.36	0.18	0.13 *			
Step 4				24.03 **	0.21	0.08
Gender	−3.79	1.94	−0.12			
Parental education	2.77	0.74	0.22 **			
Solution development and evaluation (Problem-solving)	−0.11	0.19	−0.04			
Flexibility to change the solution (Problem solving)	1.53	0.31	0.34 **			
Step 5				0.98	0.21	0.00
Gender	−3.33	1.99	−0.10			
Parental education	2.70	0.74	0.21 **			
Solution development and evaluation (Problem solving)	−0.18	0.21	−0.06			
Flexibility to change the solution (Problem solving)	1.47	0.32	0.33 **			
Self-management	0.17	0.17	0.07			
Step 6				23.11 **	0.28	0.07
Gender	−3.43	1.90	−0.11			
Parental education	2.56	0.71	0.20 **			
Solution development and evaluation (Problem solving)	0.06	0.20	0.02			
Flexibility to change the solution (Problem solving)	1.01	0.33	0.23 **			
Self-management	0.09	0.16	0.04			
Fluid nonverbal reasoning	1.72	0.36	0.28 **			
Step 7				38.14 **	0.38	0.10
Gender	−2.96	1.77	−0.09			
Parental education	1.90	0.67	0.15 **			
Solution development and evaluation (Problem solving)	0.12	0.19	0.04			
Flexibility to change the solution (Problem solving)	0.87	0.30	0.20 **			
Self-management	0.04	0.15	0.02			
Fluid nonverbal reasoning	0.76	0.37	0.13 *			
Verbal reasoning	2.08	0.34	0.37 **			

* *p* < 0.05; ** *p* < 0.01.

## Data Availability

The data are available from the author by request.
